# *In vivo* optical modulation of neural signals using monolithically integrated two-dimensional neural probe arrays

**DOI:** 10.1038/srep15466

**Published:** 2015-10-23

**Authors:** Yoojin Son, Hyunjoo Jenny Lee, Jeongyeon Kim, Hyogeun Shin, Nakwon Choi, C. Justin Lee, Eui-Sung Yoon, Euisik Yoon, Kensall D. Wise, Tae Geun Kim, Il-Joo Cho

**Affiliations:** 1 Centre for BioMicrosystems, Brain Science Institute, Korea Institute of Science and Technology (KIST), 5 Hwarang-ro 14-gil 5, Seongbuk-gu, Seoul, 136-791, Korea; 2 Department of Electrical Engineering, Korea University, 145 Anam-ro, Seongbuk-gu, Seoul, 136-701, Korea; 3 School of Electrical Engineering, Korea Advanced Institute of Science and Technology (KAIST), 291 Daehak-ro, Yuseong-gu, Daejeon, 305-701, Korea; 4 Centre for Neuroscience, Brain Science Institute, Korea Institute of Science and Technology (KIST), 5 Hwarang-ro 14-gil 5, Seongbuk-gu, Seoul, 136-791, Korea; 5 Department of Electrical Engineering and Computer Science, University of Michigan, 1301 Beal Avenue, Ann Arbor, Michigan, 48105, USA; 6 Department of Biomedical Engineering, Korea University of Science and Technology (UST), 217 Gajeong-ro, Yuseong-gu, Daejeon, 305-350, Korea

## Abstract

Integration of stimulation modalities (*e.g.* electrical, optical, and chemical) on a large array of neural probes can enable an investigation of important underlying mechanisms of brain disorders that is not possible through neural recordings alone. Furthermore, it is important to achieve this integration of multiple functionalities in a compact structure to utilize a large number of the mouse models. Here we present a successful optical modulation of *in vivo* neural signals of a transgenic mouse through our compact 2D MEMS neural array (optrodes). Using a novel fabrication method that embeds a lower cladding layer in a silicon substrate, we achieved a thin silicon 2D optrode array that is capable of delivering light to multiple sites using SU-8 as a waveguide core. Without additional modification to the microelectrodes, the measured impedance of the multiple microelectrodes was below 1 MΩ at 1 kHz. In addition, with a low background noise level (±25 μV), neural spikes from different individual neurons were recorded on each microelectrode. Lastly, we successfully used our optrodes to modulate the neural activity of a transgenic mouse through optical stimulation. These results demonstrate the functionality of the 2D optrode array and its potential as a next-generation tool for optogenetic applications.

Recently, there has been an increasing interest in brain neuromodulation due to the motivation of studying the underlying mechanisms of brain function and providing a therapeutic intervention for neurological disorders such as Parkinson’s disease, epilepsy, and chronic pain^1^,^2^,^3^,^4^,^5^,^6^,^7^,^8^. In addition to the standard electrical stimulation and neural unit recording^9^, various stimulation modalities such as chemical^10^,^11^,^12^, optical^13^,^14^,^15^, and ultrasound^16^, have been actively investigated to modulate the activity of brain circuits. Specifically, with the introduction of light-sensitive proteins and the successful incorporation of these proteins *in vivo* (*e.g.* channelrhodopsins (ChR2) and halorhodopsins (NpHR)), optical stimulation has shown a great potential as an effective and promising means of neuromodulation^17^,^18^. One of the key advantages of optical stimulation over other stimulation modalities is its cell-type-specific stimulation capability; by genetically modifying specific types of cells via the cell-type-specific expression of light-sensitive opsins such as ChR2 and NpHR, only a specific group of cells is selectively modulated (suppressed or stimulated) by light.

Microelectromechanical system (MEMS)-based neural probes, because of their capability to detect neural signals from individual neurons simultaneously, have recently been proposed as promising platforms to monitor neural activity modulated by light^14^,^15^,^19^,^20^. MEMS technology enables an integration of multiple microelectrodes in a single shank at a location of choice and thus allows single unit recording with a high spatial resolution suitable for optogenetic applications. Hence, several MEMS neural probes that are equipped with light stimulation capability have been proposed^14^,^15^,^21^. These probes incorporate light delivery systems on a single shank through various methods such as attaching a bulky optical fibre to the probe shank^14^, monolithically integrating optical waveguides^15^,^20^, and integrating light emitting diodes (LED) directly at the tip of a neural probe^21^. In addition, light delivery at multiple stimulation sites has been demonstrated by integrating the light delivery system on a two-shank probe^20^ and 3D MEMS structures^22^,^23^,^24^,^25^. However, the presented 3D multi-shank probes either lacked microelectrode arrays or were too short in length (<1 mm) to target deep-brain regions. A MEMS neural probe with the capability of delivering light to multiple sites while simultaneously recording neural signals would greatly facilitate the investigation of the neural circuits involved in brain disorders. For example, by using multi-shank neural probes to suppress other irrelevant neural circuits using halorhodopsins (NpHR), sole investigation of a neural circuit associated with a brain disorder is possible. Also, such probes can be used for pre-screening and mapping candidates of unknown brain regions by simultaneously activating multiple regions. In addition, for cases of epileptic seizure, simultaneous light stimulation at multiple sites can prevent seizure propagation. A list of other interesting biological questions that can be addressed with such neural probes but with independent light control has previously been provided^26^.

Therefore, in this work, we propose a new 2D MEMS neural probe that is monolithically integrated with optical waveguides and microelectrode arrays suitable for optogenetic applications (optrodes) in small animals. While our neural probe is a derivative of the Michigan neural probes initially developed for neural unit recording^9^ and is an extension of previously reported optrodes^13^,^27^, our fabrication method offers a major breakthrough allowing us to achieve a stress-free and thick cladding layer for these silicon-based optrodes. Specifically, our optrode offers three main advantages: 1) ability to form a thick yet embedded cladding layer, 2) U-groove structure to assist alignment between waveguide core and optical fibre, and 3) multi-shank structure (Fig. 1). To minimize the propagation loss of light, a sufficiently thick cladding layer is important because it serves as a medium for evanescent waves to sufficiently decay^28^. However, most cladding layers used for MEMS optrodes are composed of dielectric layers, while it requires a complex modification to the standard fabrication process to deposit a stress-free dielectric layer thicker than 1 ~ 2 μm^15^,^23^,^24^. The proposed structure uses a glass reflow process to readily achieve a stress-free thick cladding layer (Borofloat® 33) embedded in silicon. In addition, a thick waveguide core is achieved using SU-8, which allows for an accurate alignment of an optical fibre to the integrated waveguide core. By placing the optical fibre in a pre-defined groove, the centre of the optical fibre can be readily aligned to the centre of the SU-8 core. Lastly, the proposed structure uses multiple Y-shaped optical splitters to guide light from a single source to multiple target sites and thus enables multi-site optical stimulation with simultaneous neural signal recording.

## Results and Discussion

### Fabrication of 2D MEMS optrode array with optical waveguides

**Figure 2:**
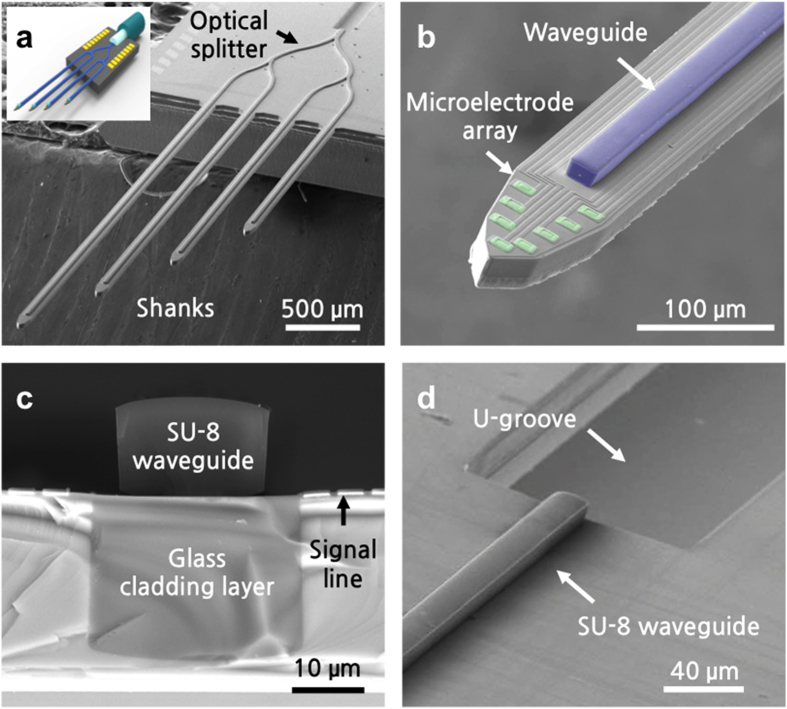
SEM images of the fabricated optrodes. (**a**) Bird’s eye view of multi-shank optrode array with an inset showing the conceptual diagram. (**b**) Tip of a single shank showing the optical waveguide core and microelectrode array. (**c**) Cross-section of the shank showing the embedded glass cladding and SU-8 waveguide core layers. (**d**) U-groove to assist alignment of optical fibre to the SU-8 waveguide core.

Using the fabrication process outlined in the Methods section, we have successfully fabricated multi-shank optrodes with four shanks, 32 recording microelectrodes (eight on each shank), and a SU-8 waveguide core (Fig. 2(a)). We observed a bending of less than 20 μm at the end of a 6-mm-long shank, which indicates that the residual stress in both the glass cladding layer due to thermal mismatch and in the thick SU-8 waveguide was minimal. The fabricated probe shank was 86-μm wide and 30-μm thick. In the present design, the lengths of the probe shanks varied from 3 to 6 mm. To target various regions in the mouse brain, we also fabricated different multi-shank designs in terms of number of shanks, lengths, and number of microelectrodes in the same run. A bird’s eye view of the scanning electron microscopy (SEM) image of the fabricated optrode shows the patterned SU-8 waveguide core with Y-shaped optical splitters on a four-shank silicon optrode (Fig. 2(a)). At each Y-shaped junction, the width of the waveguide was tapered down by 10 μm with a large bending radius (>1 mm) to minimize the bending loss^29^ (see Fig. S1 in the Supplementary Information for close-up images of our optical splitters). The close-up image of the tip of the optrode shows eight 14-μm-by-14-μm iridium microelectrodes that are located around the optical stimulation site to monitor changes in the neural activity induced by optical stimulation (Fig. 2(b)). The cross-section of the optrode illustrates the 30-μm-wide, 20-μm-thick embedded cladding layer with the 20-μm-wide, 15-μm-thick SU-8 optical core layer (Fig. 2(c)). The close-up image at the optical interface shows that the SU-8 waveguide core is aligned to the 55.5-μm-deep U-groove which is designed for placement of a 125-μm-wide optical fibre (Fig. 2(d)).

### Characterization of optical properties

**Figure 3:**
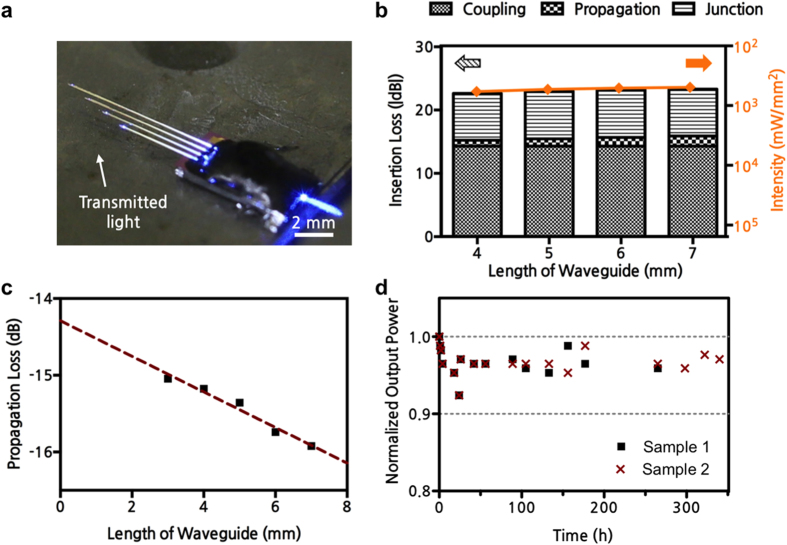
Optical characteristics of fabricated SU-8 waveguides. (**a**) Optical picture of the packaged multi-shank optrode. (**b**) Insertion loss and optical intensity (reverse y-axis) measured for different lengths of shanks with two Y-shaped optical splitters. (**c**) Insertion loss measured for different lengths of straight SU-8 waveguide cores on a test structure. (**d**) Normalized output power measured over 350 hours.

Since a sufficiently large optical power density (1 mW/mm^2^) is required to activate channelrhodopsin-2 (ChR2)^18^, it is important to measure the output power and to characterize the optical properties of the proposed Y-shaped SU-8 waveguide structure. To provide an optical interface, an optical fibre connected to a laser source was placed in the U-groove (see Methods for details on device packaging and measurement systems). Light transmitted from a 475-nm blue laser source through an external multimode optical fibre was successfully guided to each stimulation site through the waveguide cores of varying lengths (Fig. 3(a)). When the input power (*i.e.* the power measured at the end of the optical fibre) was 32 mW, the output power measured at the end of shanks with 4, 5, 6, and 7-mm-long waveguides were 175, 161, 153, and 149 μW, respectively. The corresponding optical power densities at these four stimulation sites, which were in the range of 500 ~ 583 mW/mm^2^, are all approximately five hundred times larger than the intensity required to activate ChR2 (Fig. 3(b)). To estimate the reliability of our fabrication process, we also measured the output power densities of six different multi-shank optrodes with identical waveguide structures (*i.e.* a 6-mm-long shank with 7-mm-long waveguides integrated with Y-shaped optical splitters). The mean output power density of the 6-mm-long shanks from the six different devices was 500 mW/mm^2^, with a standard deviation of 3.47%.

In our light delivery scheme, there are three main sources of optical loss that can be attributed to the overall insertion loss: coupling loss (between the external optical fibre and the SU-8 waveguide core), propagation loss (through the SU-8 waveguide), and junction loss (at the Y-shaped optical splitters). First, we used the cutback method^30^ to estimate the coupling and propagation loss through the 20-μm-wide, 15-μm-thick SU-8 waveguide core on the thick glass cladding layer. Specifically, a test structure consisting of different lengths of straight SU-8 waveguide cores was fabricated and the output power at the end of each waveguide core was measured (Fig. 3(c)). Based on these measurements from a single test device, we estimated the propagation loss at −0.23 dB/mm by computing the slope and the coupling loss at −14.3 dB by finding the y-intercept through extrapolation. This propagation loss is mainly due to natural attenuation in SU-8 which depends highly on the wavelength^29^,^31^,^32^. Based on these transmission properties of single SU-8 waveguide cores and our measurements from the multi-shank optrode with two Y-shaped splitters, we estimated the mean junction loss at −7.45 dB (Fig. 3(b)). Therefore, the optical loss at each Y-shaped junction is approximately −3.72 dB. By using a large bending radius (>1 mm), the bending loss was minimized^29^. Although the coupling loss is difficult to reduce, the junction and propagation losses can be further reduced by optimizing the shape of the optical splitter and reducing the surface roughness of the waveguide core, respectively.

Despite the additional optical loss due to the optical splitter, the presented optrode exhibits an output intensity that is approximately ten times larger than that of previously reported optrodes with SU-8 waveguides^20^,^33^. One of the key differences between our optrode and the previously reported optrodes is the thick cladding layer. The cladding layer is important because it serves not only as a boundary that causes total internal reflection and confines the light in the waveguide, but also as a medium for evanescent waves to sufficiently decay. Thus, if the cladding layer were not sufficiently thick, the amplitude of the evanescent waves at the cladding and outer neighbouring region would be large, causing significant attenuation^28^. For example, in our system, a glass cladding layer of at least 3.2 μm thickness is required so that the normalized amplitude of the evanescent wave will be smaller than 1% at the boundary between the cladding and the bottom silicon^34^.

Since the key advantage of optical stimulation is local stimulation, it is important that the delivered light at the end of SU-8 waveguide be spatially confined. We simulated the intensity based on the 1/*d* scattering model for *d *< 1 mm^35^, the mean scattering coefficient of blue light in the brain^26^, and the standard geometric dispersion model. We estimated that light decayed below the activation threshold power density (1 mW/mm^2^) at approximately 260 μm from the end of the SU-8 waveguide and that all microelectrodes were illuminated with a sufficient light intensity for activation (see Fig. S2 in the Supplementary Information for models, equations, and intensity map). This rapidly decaying profile suggests that not only is the light spatially confined through the small cross-section of the SU-8 waveguide, as expected, but also that a sufficient light intensity is delivered to neurons near the recording sites. However, during the optical characterization, we observed apparent light leakage along the SU-8 waveguide (Fig. 3(a)). Since our goal is to stimulate local sites (≤200 μm), it is essential that this light leakage be sufficiently low as to not optically stimulate neighbouring neurons. If we assume for the worst-case estimation that 100% of the propagation loss is due to light leakage (despite the fact that the propagation loss is mainly due to natural attenuation through the SU-8 material), we can estimate the power density of light through the sidewalls at 0.21 mW/mm^2^ when the input power is 32 mW. This estimated intensity is approximately a thousand times smaller than the output intensity at the stimulation site and is below the activation threshold intensity (see Fig. S3 in the Supplementary Information for more detail). If the leakage power at the sidewall of our optrodes is not sufficiently small, the input power can be further reduced because the output power at the waveguide is still five hundred times larger than the activation threshold power density (1 mW/mm^2^).

The long-term reliability of the optrode is also critical for chronic applications that require *in vivo* experiments over several weeks^36^. For effective study of behaviour-related or disease-related neural signals, the long-term monitoring of neural activity through a fixed implanted optrode is essential. A representative long-term study on the optical properties of polymer-based waveguide structures has been reported but SU-8 was used as a cladding layer rather than as a core layer; moreover, only approximately 80 days of long-term data were shown^37^. We conducted an accelerated life testing^38^ at an elevated temperature to assess the long-term reliability of our probes. Two packaged optrodes were immersed in a saline solution to simulate the *in vivo* environment and the saline solution was maintained at 90 °C for the entire period of accelerated life testing. Because its ionic contents and osmolarity are similar to those cerebrospinal fluid (CSF), saline solution was used as a model; in addition, 99% of both saline solution and CSF are water based^39^. While inspecting for any mechanical damage on the probe or the package over time, we examined the long-term effect of the 90 °C saline solution on the optical property by measuring the output optical power at the stimulation site. An output power of more than 90% of the initial optical power was maintained for soak times of 350 hours, which is equivalent to six months at a typical body temperature of 37 °C (Fig. 3(d)). Therefore, our long-term reliability tests show that the expected lifetime of our optrode for optical simulation is adequate for chronic experiments. The mode of failure for our probe was delamination; after 350 hours, the SU-8 waveguide core detached from the cladding layer of the optrode. Potential methods to improve the adhesion between the SU-8 core and the oxide cladding layers include increasing the width of the SU-8 waveguide to increase the bonding area and optimizing the baking condition during the SU-8 patterning process to minimize the residual stress. In an additional experiment, we delivered blue light with a duty cycle of less than 50% for 3 hours *in vivo* and observed no degradation of the SU-8 waveguide due to the light delivery.

While dielectric layers such as silicon oxynitride have been common choices for the waveguide material in silicon neural probes^15^,^24^, we chose SU-8 because this material allows a thick waveguide to be readily fabricated without stress compensation^13^. A thicker waveguide allows for easier vertical alignment to the optical fibres. Despite the high visible light transmission (VLT), a main constraint of using dielectric layers is thickness. Stress compensation of a deposited dielectric layer with a thickness greater than 3 μm depends heavily on the condition of the fabrication equipment^15^. On the other hand, SU-8 exhibits not only a high VLT (90 ~ 95%), but also offers several additional advantages such as low surface roughness, a wide range of thickness, and simple fabrication steps. Previously, the long-term reliability of SU-8 waveguides *in vivo* was hypothetically questioned due to high water absorption rates^15^; however, our work demonstrated otherwise through long-term reliability tests.

### *In vivo* neural recordings and optical modulation

We packaged our 2D optrode on a customized printed circuit board and provided an electric interface to one shank out of four to measure neural activity. The average impedance of 6 of the working microelectrodes out of 8 was below 1 MΩ at 1 kHz, which is low enough to measure *in vivo* neural signals (see Methods for details on EIS measurements and *in vivo* procedures). We conducted two separate acute *in vivo* experiments: one with a wild-type mouse (B6 mouse; male; 8 weeks) as a control to study the artefacts and one with a transgenic Thy1-ChR2-YFP (yellow fluorescent protein) mouse to monitor the light modulation. For both mice, neural spike signals from the hippocampus (stratum pyramidale, CA1) of the anesthetized mice were measured under the same light stimulation conditions. For the wild-type mouse, we detected spontaneous neural spike signals with noise levels of approximately ±25 μV. Under light stimulation, the noise level almost doubled (±50 μV), while we did not observe any significant increase in the number of spikes that were larger than the noise level (either neural signals or artefacts).

**Figure 4:**
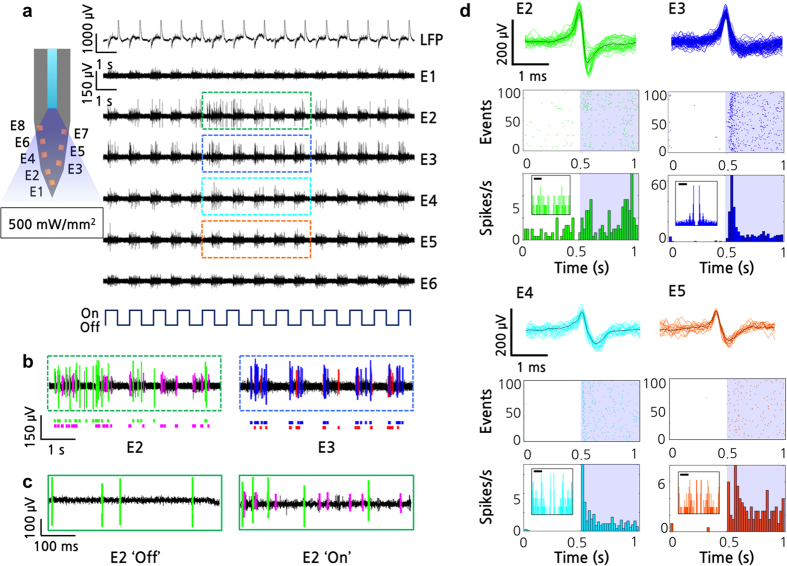
*In vivo* experimental results with light stimulation. (**a**) *In vivo* neural spike signals from hippocampus of a transgenic mouse (stratum pyramidale, CA1) measured using six microelectrodes during light stimulation with LFP shown on top. A train of light pulses with a frequency of 1 Hz and a duty cycle of 50% was applied. (**b**) Raster plot of sorted neural signals on E2 and E3. (**c**) Close-up transient plots of neural signals detected from E2 during ‘off’ and ‘on’ cycles. (**d**) Sorted neural spike signals from E2, E3, E4, and E5 and their corresponding raster plots and peristimulus time histograms (PSTH) of 100 events (*i.e.* pulses). The inset shows the autocorrelograms of the sorted signals (bar: 20 ms).

For the transgenic mouse, neural spike signals were successfully detected from 6 microelectrodes integrated on a single shank (Fig. 4(a)). With a low background noise level of approximately ±25 μV, distinguishable spontaneous neural spikes from individual neurons were recorded on each electrode. Examples of these spontaneous signals are the ones observed at the ‘off’ cycle during light stimulation (Fig. 4(c)). Upon application of a train of blue light pulses (1 Hz with a 50% duty cycle), neural activities were synchronized with the light pulses; neural activities detected on electrodes 2, 3, 4, and 5 (*i.e.* E2, E3, E4, and E5) were found to significantly increase during light stimulation (Fig. 4(a)). At every onset and offset of light stimulation, we observed large fluctuations in the local field potential (LFP).

We analysed a segment of recorded neural signals and sorted out neural spikes from different neurons using a custom MATLAB^©^ sorting algorithm (Fig. 4(b)). To prevent misidentification of the artefacts as neural spike signals, we sorted only the spikes with amplitudes larger than the increased noise level in the wild-type case (*i.e.* ±50 μV) by setting the amplitude threshold to be 65 μV. In addition, we plotted autocorrelograms to observe the refractory period. The signal analysis shows that at least one distinctive set of neural spike signals was detected for each of E2, E3, E4, and E5. For example, one set of neural spike signals was sorted out from signals detected from E2 and E3; these signals are shown in green and blue, respectively. These two sets of signals show different amplitudes and shapes, which implies that neural spike signals from two different neurons were recorded. We observed similar results for E4 and E5. For the cases of E1 and E6, we do not claim any changes in neural activity because the amplitudes of the spikes observed during light stimulation were comparable to the increased noise level of the wild-type case. Thus, our future work will include optimization of the recording setup to minimize the noise during the light optical stimulation, so that our system can distinguish a larger number of neural spike signals.

The raster plots (*i.e.* timestamps) shown below the transient plots indicates that the timestamps of the spikes from the sorted signals do not overlap, which also implies that the neural signals were successfully sorted. The raster plots of these sorted neural signals from 100 cycles (*i.e.* events) are vertically stacked and their corresponding peristimulus time histograms (PSTHs) are plotted. These plots confirm the dramatic increases in the number of neural spikes during the 0.5-s optical stimulation periods (Fig. 4(d)). Specifically, neurons with almost no activity when light was off fired at much higher rates upon light stimulation, which confirms that neurons in the hippocampus of the transgenic mouse (Thy1-ChR2-YFP) were successfully stimulated. These *in vivo* results demonstrate that our optrode is capable of modulating and recording the neural activities of certain groups of neurons through optical stimulation. We verified that the existence of ChR2 via the histology of the sacrificed mouse brain at the end of the *in vivo* unit recording experiments.

Although concurrent light delivery to all stimulation sites is the main functionality of our optrode, one limitation of our proposed design is the lack of independent light control of each stimulation site. For more interesting neuroscience applications, independent stimulation of different stimulation sites would be an essential addition to our current design; this will be part of our future work. For example, a close investigation of the target region is possible by silencing neighbouring regions with yellow light while stimulating the target region with blue light. A crude but simple method of enabling independent light delivery is to fabricate multiple grooves to allow multiple external light sources. Another method is to use one or two light sources with optical switches based on optoelectronic techniques such as thermo-optical switching. Direct addition of light sources at the tip of the shank is another method to allow independent light delivery^18^,^40^. However, since direct addition at the tip increases the thickness of the neural probes (>200 μm), integration of a laser diode chip at the tip^20^ or implementation of a high-density 3D structure with an external light control system^24^ are more promising alternative solutions.

## Conclusions

Our MEMS optrode enables simultaneous optical stimulation of different brain regions by integrating a SU-8 waveguide structure and optical splitters on a silicon optrode array. The integrated microelectrode array also enables simultaneous monitoring of neural activities modulated by optical stimulation. In addition, we explored the long-term stability of the probe for chronic applications. This probe is a promising tool for studying the brain by controlling brain circuits involved in brain diseases; more specifically, the probe works by stimulating and suppressing specific types of neurons and by simultaneously recording neural signals in multiple regions. This new capability should play a key role in functionally mapping the brain as well as in understanding and treating various disorders. Since the functionality of one shank from our multi-shank optrode is confirmed, our future work will include utilizing a multi-shank structure to examine and control disease-related brain circuits and integrating an optical switch with the optical waveguide to extend potential applications.

## Methods

### Fabrication of optrode array integrated with an SU-8 waveguide structure

**Figure 5:**
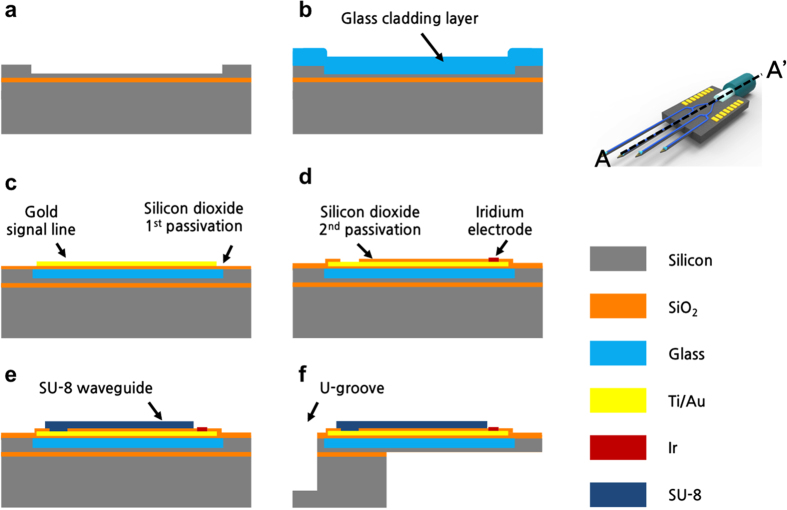
Fabrication process flow along A–A’. (**a**) Groove formation. (**b**) Anodic bonding of a glass wafer and thermal reflow. (**c**) Removal of the unwanted glass and deposition of an insulation layer. (**d**) Signal line and microelectrode array patterning. (**e**) SU-8 waveguide patterning. (**f**) Release.

We started with a 4-inch silicon-on-insulator (SOI) wafer with a 30-μm-thick top silicon and 0.7-μm-thick buried-oxide layer (BOX). First, a groove was patterned on the SOI wafer; it was etched 20-μm-deep using deep reactive-ion etching (DRIE) to help form the cladding layer in subsequent steps (Fig. 5). Then, the SOI wafer was anodically bonded to a 500-μm-thick borosilicate glass wafer in vacuum. Next, this glass was thinned down to 100 μm by chemical-mechanical polishing (CMP) and then reflowed at 800 °C for 2 h in a furnace^19^,^41^,^42^. The groove was completely filled with the reflowed glass. During the thermal cycle, the thermal mismatch between the borosilicate glass and the silicon was the greatest at the cooling step from 800 °C to 550 °C^43^. However, the stress induced from this thermal mismatch is only on the order of several MPa. We observed no significant bending on any of the neural probes that were fabricated using the glass reflow process and thus stress characterization and compensation were not necessary. The reflow process allows the thickness of the cladding layer to be accurately controlled with an upper limit set by the desired final probe thickness. The lower limit of the thickness of the cladding layer is approximately 5 μm; this value was determined by the ±2-μm variation in the consequent CMP process. With a 5 μm-thick glass cladding layer, the thickness of our optrode can be further reduced to 10 ~ 15 μm. After the unnecessary glass was removed using CMP, the planarized glass-in-silicon wafer was available for further fabrication processes.

After depositing a 300-nm-thick silicon dioxide (SiO_2_) insulation layer using plasma-enhanced chemical vapour deposition (PECVD), adhesion layers of 300-nm-thick gold and 20-nm-thick chromium were deposited and patterned using a lift-off process to form 3-μm-wide signal lines with 3-μm spacing. Minimal achievable line width and line spacing using the lift-off process were both approximately 2 μm. These lines were protected by a 400-nm-thick SiO_2_ layer deposited using PECVD; then, contact areas were opened. Iridium (Ir) microelectrodes were formed by depositing 20-nm-thick titanium (Ti) and 150-nm-thick Ir layers, followed by the lift-off process. Next, a 15-μm-thick SU-8 layer was spin-coated and patterned to form the waveguide core. After patterning the SU-8 waveguide core on the cladding layer, a U-groove for fibre placement was patterned using DRIE. SU-8 waveguides were protected throughout the consequent steps to preserve the low surface roughness. To allow for a sufficient alignment margin, the gap between the proximal end of the waveguide and the end of glass cladding layer was 5 μm. Similarly, the gap between the end of the glass cladding layer and the end of the groove was 5 μm. The minimum alignment margin in our process was 2 μm. The depth of the groove was precisely controlled using DRIE to achieve accurate alignment between a 125-μm-wide multimode optical fibre and the SU-8 waveguide core. The groove height of 55 μm was etched such that the centre of the 125-μm-wide optical fibre was vertically aligned to the centre of the 15-μm-high SU-8 waveguide. First, the 30-μm-top-silicon layer was removed using DRIE with the BOX layer as an etch stop. Then, the 0.7-μm-BOX layer was removed using RIE with a non-uniformity variation of 5%; the remaining 24.3-μm silicon was removed using DRIE with the same uniformity variation. Thus, the groove height was controlled within ±1.25 μm.

Finally, the probe was released from the backside using DRIE. The overall shape of the probe was patterned on the front and etched using DRIE. Then, with the front side of the wafer protected by a photoresist (AZ® 9260, MicroChemicals GmbH, Germany), the probe shanks were released using the standard release process developed for SOI wafers^44^. Specifically, the backside of the SOI wafer was patterned and etched to the BOX layer using DRIE to release the probe shanks. The remaining BOX layer was etched from the backside using RIE to completely isolate the probe shanks from the substrate.

### Device packaging

First, the device was packaged to provide an electric interface. The optrode was glued on a custom printed circuit board (PCB) using a fast curing epoxy (5-min Epoxy Resin, ITW Devcon, Riviera Beach, FL). The electrode pads on the probe were wire-bonded to the pads on the PCB, which provided electric connections to the Neuralynx recording system through a 16-channel connector (Omnetics Connector Corporation, Minneapolis, MN). Although 32 channels were available from our 4-shank neural probes, we only wire-bonded 8 pads because our *in vivo* experiment was designed for a single shank test. Two wirebonds were broken during the assembly and thus 6 microelectrodes were available.

Next, our optrodes were packaged with an optical fibre to transmit light from the light source to stimulation sites. We used a multimode optical fibre (GIF50, Thorlab, Newton, NJ, USA) that was composed of a core and cladding layer that were 50 and 125 μm in diameter, respectively. The end of the fibre was terminated with a FC/PC connector and connected to a light source (ADR-2301, RGBlase LLC, CA, USA). The other end of the optical fibre was placed on the pre-defined U-groove of the optrode. After mounting the electrically packaged optrode on a mechanical xyz-stage, we manually aligned the fibre in the lateral direction under a microscope with the groove as the guide. Without manual alignment, the worst-case alignment mismatch between the centre of the fibre and that of the waveguide was approximately 15.5 μm. Since the diameter of the optical fibre was larger than the cross section of the SU-8 waveguide core (width: 20 μm; thickness: 15 μm), alignment was straightforward. Then, for index matching, UV-curable epoxy (NOA 148, Norland Products Inc., Cranbury, NJ, USA, *n*: 1.48) was applied to fill the gap between the optical fibre and the SU-8 waveguide core. As a final step, the body of the optrode was covered with a biocompatible black epoxy (EPO-TEK 320, Epoxy Technology, Inc., Billerica, MA, USA) to block light that was lost through coupling.

### Experimental setup to measure optical output power

To characterize the light intensity from each stimulation site, a photodetector (918D, Newport Inc., Irvine, CA, USA) connected to an optical power meter (1936-R, Newport Inc., Irvine, CA, USA) was placed near the end of the optrode to measure the light intensity. The fluctuation in output measurement was within +/− 0.002 mW.

### Electrochemical impedance spectroscopy (EIS)

The electrode-electrolyte interface of the iridium microelectrodes was characterized using the two-electrode cell configuration. The optrode (a working electrode) and an Ag/AgCl electrode (a reference electrode) were immersed in 1× phosphate-buffered saline (PBS, an electrolyte). Electrochemical impedance spectroscopy (EIS) was performed over a frequency range of 10 Hz to 10 kHz using a commercial impedance analysis system (nanoZ, Neuralynx, Montana, USA).

### *In vivo* experimental setup

All mice experiments conducted for this study were approved by the Korea Institute of Science and Technology (KIST, Seoul, Korea) and were performed in accordance with the ethical standards stated in the Animal Care and Use Guidelines of KIST. An adult male wild-type mouse (B6; 8 weeks) and an adult female transgenic mouse (B6 Thy1-ChR2-YFP; 10 weeks) were used. The transgenic mouse was expected to contain ChR2 transgenic mouse lines (Thy1-ChR2-EYFP lines 9 and 18) with ChR2 fused with enhanced YFP (EYFP) expressed in a subset of neurons throughout the nervous system^45^,^46^. The mice were anesthetized with 2% avertin (400 mg/kg, intraperitoneal injection). After placing the mice on a stereotaxic device (Kopf, USA), the skin was incised and the skull was drilled over the target area of the hippocampus (Anterior - Posterior (AP): −1.6, Medial – Lateral (ML): −1.6 for the wild-type; AP: −1.8, ML: −1.7 for the transgenic mouse) based on the atlas of Paxinos and Franklin^47^. The packaged optrode was inserted through the pia and lowered slowly down to the target region (Dorsal – Ventra (DV): −1.43 for the wild-type; DV: −1.8 for the transgenic mouse). After reaching the target region, we waited 30 minutes for stabilization.

The laser source (ADR-2301, RGBlase LLC, CA, USA) was used with an optical chopper to modulate the light. A square wave with a period of 1 s and a duty cycle of 50% was applied. Neural spike signals from six microelectrodes were recorded using Neuralynx (Digital Lynx 4SX, Neuralynx, MT, USA); the raw signals were filtered through the Neuralynx software with two sets of band-pass filter parameters (0.3 Hz–6 kHz for LPF and 300 Hz–6 kHz for individual neural spike signals).

## Additional Information

**How to cite this article**: Son, Y. *et al. In vivo* optical modulation of neural signals using monolithically integrated two-dimensional neural probe arrays. *Sci. Rep.* **5**, 15466; doi: 10.1038/srep15466 (2015).

## Supplementary Material

Supplementary Information

## Figures and Tables

**Figure 1:**
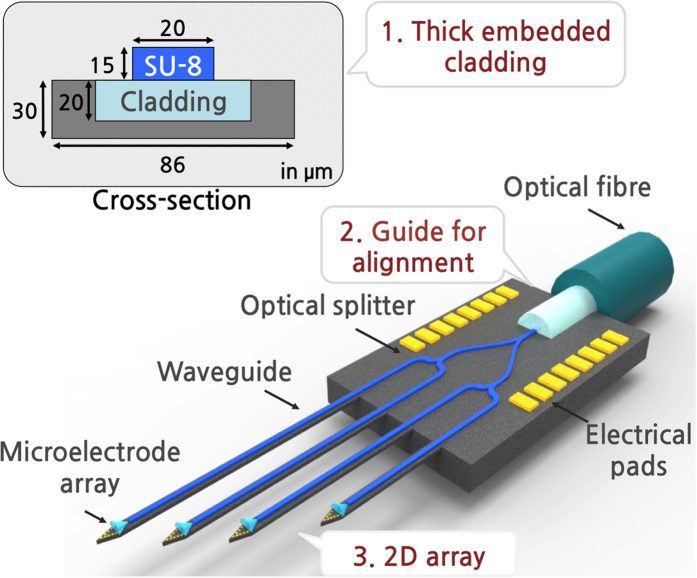
Schematic of the proposed multi-shank (2D) optrode array for optogenetic applications. The inset shows the cross-section of a probe shank.
