# Supplementary material for: In vivo optical modulation of neural signals using monolithically integrated two-dimensional neural probe arrays
Source: Sci Rep. 2015 Oct 23;5:15466. doi: 10.1038/srep15466 (PMC4616027; doi:10.1038/srep15466)
Supplement: Supplementary Information [file srep15466-s1.pdf]

Supplementary Information

In vivo optical modulation of neural signals using monolithically integrated two-dimensional neural probe arrays

Yoojin Son[a,b,†], Hyunjoo Jenny Lee[a,c,†], Jeongyeon Kim[d], Nakwon Choi[a], C. Justin Lee[d], Eui-Sung Yoon[a], Euisik Yoon[e], Kensall D. Wise[e], Tae Geun Kim[b], and Il-Joo Cho[a,]*

[a.] Centre for BioMicrosystems, Brain Science Institute, Korea Institute of Science and Technology (KIST), Hwarangro 14-gil 5, Seongbuk-gu, Seoul, 136-791, Korea

[b.] Department of Electrical Engineering, Korea University, 145 Anam-ro, Seongbuk-gu, Seoul, 136-701, Korea

[c.] School of Electrical Engineering, Korea Advanced Institute of Science and Technology (KAIST), 291 Daehak-ro, Yuseong-gu, Daejeon, 305-701, Korea

[d.] Centre for Neuroscience, Brain Science Institute, Korea Institute of Science and Technology (KIST), Hwarangro 14-gil 5, Seongbuk-gu, Seoul, 136-791, Korea

[e.] Department of Electrical Engineering and Computer Science, University of Michigan, 1301 Beal Avenue, Ann Arbor, Michigan, 48105, USA

[*] E-mail: ijcho@kist.re.kr

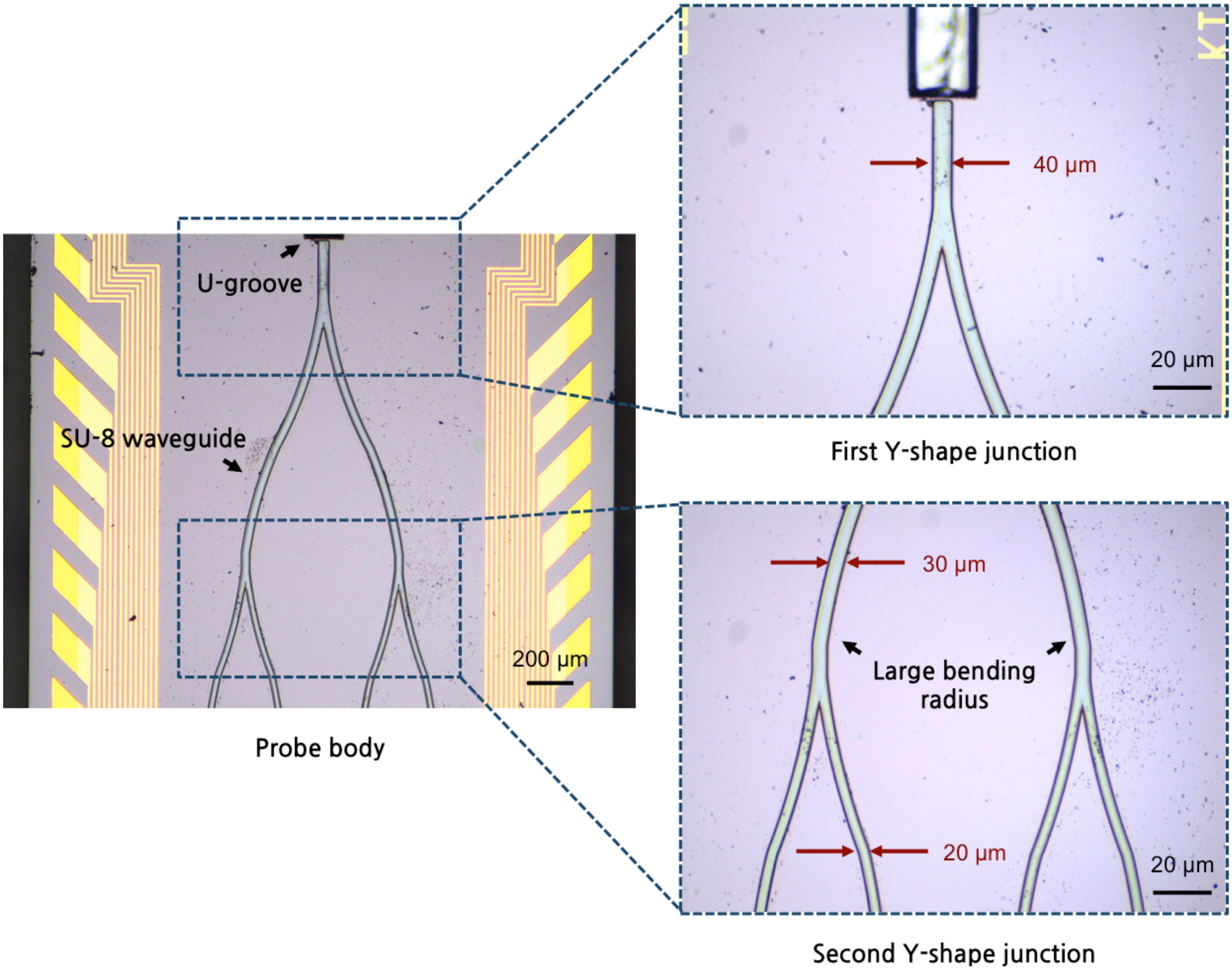

Figure S1. Optical images showing the Y-shape optical splitters.

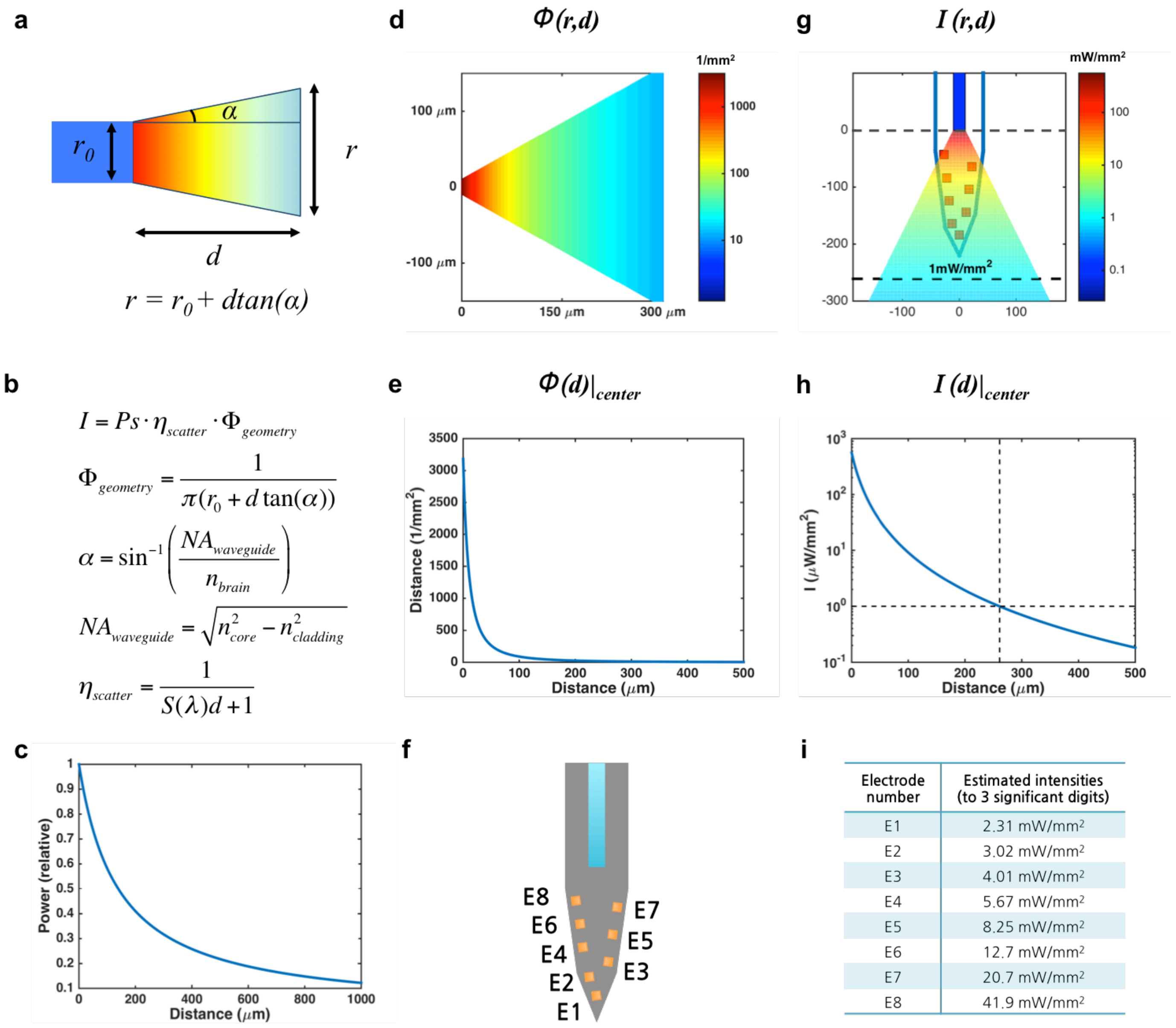

Electrode number	Estimated intensities (to 3 significant digits)
E1	2.31 mW/mm²
E2	3.02 mW/mm²
E3	4.01 mW/mm²
E4	5.67 mW/mm²
E5	8.25 mW/mm²
E6	12.7 mW/mm²
E7	20.7 mW/mm²
E8	41.9 mW/mm²

Figure S2. Estimation of optical intensities in brain (a) A schematic the triangular light propagated along the axial distance, *d*, from the waveguide (blue box with a radius of r_o). Since our waveguide was a rectangular structure, an equivalent radius was computed (*i.e.* r_o = 9.77 μm). (b) *I* is intensity, *Ps* is the source power, $\eta_{scatter}$ is 1/d scattering model for $d < 1$ mm to estimate power attenuation (Aravanis et al, 2007 and Stark et al, 2012), $\Phi_{geometry}$ is geometric dispersion of light inversely proportional to area of cone cross-section, *NA* is the numerical aperture of the waveguide, *n* is refractive index, and *S* is mean scattering coefficient at certain wavelength (*S*(blue) = 7.2, Stark, 2012). Note, $\Phi_{geometry} = 0$ when $r > r_o + d\tan(\alpha)$. (c) Estimated relative power attenuation, $\eta_{scatter}$, over *d*. (d) $\Phi_{geometry}$ as a function of *r* and *d*. (e) $\Phi_{geometry}$ along

the fibre centre. (f) Numbering of 8 microelectrodes. (g) Light intensity map with a source (blue light, 175 μW at the end of waveguide) as a function of *r* and *d*. (h) Intensity along d at the fibre centre. (i) Summary of the estimated intensity at each microelectrode.

a

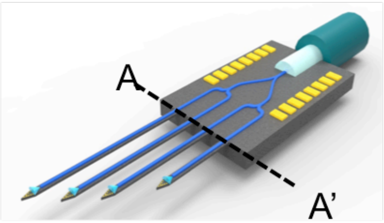

b

	Loss (dB)	Input (mW)	Output (mW)
1. Coupling	-14.3	32	1.19
2. Junction	-7.45	1.19	0.21
3. Propagation (after 1 mm)	-0.23	0.21	0.20

c

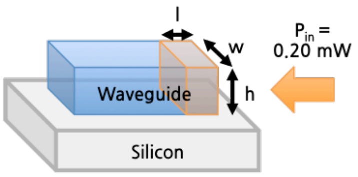

d

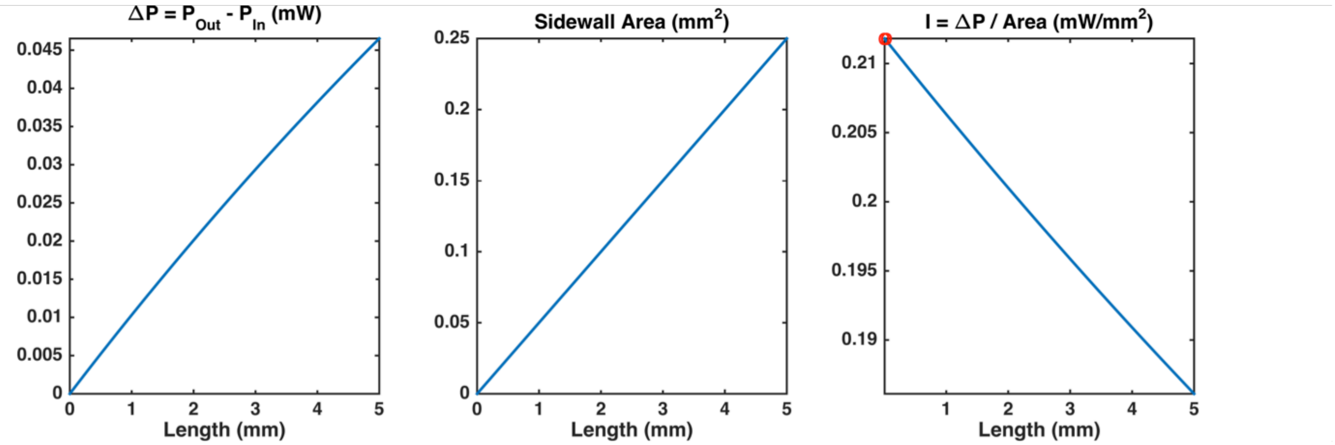

Figure S3. Estimation of leakage intensities A worst-case estimation of the light lost through the sidewall. (a,b) First, we compute the input power at A-A' starting with an input power of 32 mW. (c) Then, we assume 100% of the propagation loss is due to the sideway leakage. To compute the intensity, the sidewall area must be defined which depends on the length along the waveguide. (d) Numerical computation shows that the worst-case intensity of ~0.21 mW/mm^2 is estimated at A-A'.
